# A hybrid neural ordinary differential equation model of the cardiovascular system

**DOI:** 10.1098/rsif.2023.0710

**Published:** 2024-03-20

**Authors:** Gevik Grigorian, Sandip V. George, Sam Lishak, Rebecca J. Shipley, Simon Arridge

**Affiliations:** ^1^ Department of Mechanical Engineering, University College London, WC1E 6BT London, UK; ^2^ Department of Computer Science, University College London, WC1E 6BT London, UK; ^3^ Department of Physics, University of Aberdeen, AB24 3FX Aberdeen, UK

**Keywords:** cardiovascular system, ordinary differential equations, time series, neural network, symbolic regression

## Abstract

In the human cardiovascular system (CVS), the interaction between the left and right ventricles of the heart is influenced by the septum and the pericardium. Computational models of the CVS can capture this interaction, but this often involves approximating solutions to complex nonlinear equations numerically. As a result, numerous models have been proposed, where these nonlinear equations are either simplified, or ventricular interaction is ignored. In this work, we propose an alternative approach to modelling ventricular interaction, using a hybrid neural ordinary differential equation (ODE) structure. First, a lumped parameter ODE model of the CVS (including a Newton–Raphson procedure as the numerical solver) is simulated to generate synthetic time-series data. Next, a hybrid neural ODE based on the same model is constructed, where ventricular interaction is instead set to be governed by a neural network. We use a short range of the synthetic data (with various amounts of added measurement noise) to train the hybrid neural ODE model. Symbolic regression is used to convert the neural network into analytic expressions, resulting in a partially learned mechanistic model. This approach was able to recover parsimonious functions with good predictive capabilities and was robust to measurement noise.

## 1. Introduction

The development of biomechanical mathematical models is a vibrant area of research which aims at enhancing our understanding of human physiology, while also providing a tool to make clinically relevant predictions. Models of the human cardiovascular system (CVS) are examples of such physiological models. The simplest versions of these are often referred to as lumped-parameter or pressure-volume models, whereby the CVS is divided into distinct chambers which capture the main dynamics of the system. These lumped-parameter models contain no spatial information and are usually mathematically prescribed as a system of ordinary differential equations (ODEs). Higher fidelity models which incorporate spatial information are solved using other numerical methods, for example finite-element analysis (FEA) [1–4], and can provide very detailed and accurate representations of the underlying dynamics. However, challenges remain around assigning model parameter inputs, for example muscle fibre orientations or material parameters.

We restrict our ongoing discussion to temporal ODE models of the CVS, early examples of which are inspired by electrical circuits [5,6]. Multi-compartment alternatives such as the seminal models by Arthur C. Guyton based on pig experiments conducted in the 1950s and 1960s, describe the CVS in more detail [7]. Simplified versions of these frameworks which are still capable of capturing the major governing dynamics of the CVS (and sometimes also the respiratory system) are more commonly employed [8–10]. Extrapolating these models to predict the dynamics of the human CVS is highly challenging, not least because it is difficult to directly measure variables such as blood flow and pressure inside a living human heart, meaning that the models are rarely parametrized against human data. Despite this, such models may be extremely useful in understanding human health and making predictions about the evolution of disease.

An important characteristic of the dynamics of the CVS is the interaction between the left and right ventricles of the heart, often referred to as ventricular interaction (VI). This interaction is governed by the effects of the septum (a muscular membrane separating the left and right ventricles) and the pericardium (a passive fibrous sac that encapsulates the entire heart). Capturing VI within these simplified models (as in [9]) often involves the calculation of the septum free wall volume (*V*_spt_), given by the solution to a complex nonlinear equation with no analytic solution. Hence, a numerical root-finding algorithm such as the Newton–Raphson method must be used. Due to this added complexity, various alternative approaches to modelling VI have been proposed. In [10], the problem is simplified by employing a linearization, while in [11], a more accurate (but more complex) local linearization is used. VI is modelled in a different way in [12], where the volume in the septum is defined as a linear function of the left and right ventricular pressures. There are also a number of cases where VI is left out entirely [13–16].

Within the field of scientific machine learning, recent work has demonstrated the possibility of developing partially learned models, sometimes referred to as ‘grey box’ models, wherein certain components of a mathematical (or ‘white box’) model are set to be governed by a learned (or ‘black box’) system. These partially learned systems can take a wide range of structural forms [17–21]. In the context of dynamical systems, universal differential equations (UDEs) [22] are an example of a partially learned system, where specific terms in a system of ODEs or partial differential equations (PDEs) are replaced with a neural network. Data are then used to train the model such that the network captures the dynamics missing from the system. This approach allows for an inference step, where the trained neural network embedded in the system equations can be regressed down to mathematical expressions. This technique has been applied successfully to simple dynamical systems such as the Lotka–Volterra equations [22] as well as more complex systems [23–26]. The architecture of this method can also be thought of as a hybrid neural ODE and is hereafter referred to as such.

In this work, we investigate the application of a hybrid neural ODE to the closed-loop lumped parameter model of the CVS from [9]. Specifically, we allow VI in this system to be governed by a neural network, providing an alternative means of modelling these dynamics. This hybrid neural ODE is trained using synthetic time-series data (with various amounts of added measurement noise) generated by simulating the original model as outlined in [9]. We subsequently use symbolic regression (SR) [27] to regress the trained neural network back into symbolic form, allowing for the potential discovery of a more parsimonious function for the septum free wall volume. Converting the trained network to mathematical expressions results in a more interpretable, partially learned mechanistic model. The predictive capabilities of both the trained hybrid neural ODE (before SR) and the partially learned mechanistic model are examined.

## 2. Methodology

An overview of the lumped parameter model of the CVS is given in §2.1 and the description of the hybrid neural ODE based on this model is presented in §2.2. All simulations were carried out in Julia [28], using the Tsit5 ODE solver. Due to the discontinuities in the dynamics (as a result of the opening/closing of the valves in the heart) a maximum step size of 10^−2^ was enforced. Furthermore, to achieve accurate (non-negative) flow dynamics, an absolute tolerance of 10^−7^ and a relative tolerance of 10^−4^ were used.

### 2.1. Model of cardiovascular system

**Figure 1.**
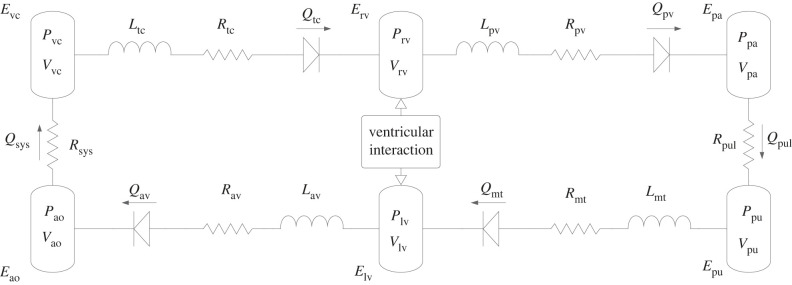
Schematic of the closed-loop model of the cardiovascular system. The governing equations for the flows (*Q*), volumes (*V*) and pressures (*P*) are defined in appendix A, while the resistances (*R*), inertial effects (*L*) and the elastances (*E*) are parameters defined in table 3.

The model, as shown in figure 1 follows established approaches in the literature [9,10,29]. The CVS is divided into the following six elastic pressure-volume chambers:

— Left ventricle (lv)
— Right ventricle (rv)
— Vena cava (vc)
— Aorta (ao)
— Pulmonary artery (pa)
— Pulmonary vein (pu)

In figure 1, the nodes represent the pressure-volume chambers. Since this is a lumped parameter model, each of these chambers captures the behaviour of the unit it contains (and this is not modelled at finer granularity). For example, the pulmonary artery chamber encapsulates the elastance of the main arteries feeding the lungs.

These six chambers are connected in series by resistances, inductors, and diodes. The resistances (*R*) capture the pressure drop of the blood as it flows between chambers, the inductors represent the inertial effects (*L*) of the blood as it flows in and out of the ventricular chambers, and the diodes represent the heart valves. Each chamber also has an elastance (*E*), with the ventricles being dynamically elastic (since they act as pumps) and the remaining chambers being passively elastic. The flows are labelled *Q*, the volumes are labelled *V* and the pressures (intermediate quantities in the model) are labelled *P*. The effects of VI, systemic circulation (sys) and pulmonary circulation (pul) are also captured.

The model consists of 10 state variables; the volumes in each of the six elastic chambers listed above, as well as the four flows for which inertial effects are included. Thus, the 10 state variables are [*Q*_mt_, *Q*_av_, *Q*_tc_, *Q*_pv_, *V*_lv_, *V*_rv_, *V*_ao_, *V*_vc_, *V*_pa_, *V*_pu_]. Here, *Q*_mt_, *Q*_av_, *Q*_tc_ and *Q*_pv_ are the flows through the mitral, aortic, tricuspid and pulmonary valves, respectively.

**Figure 2.**
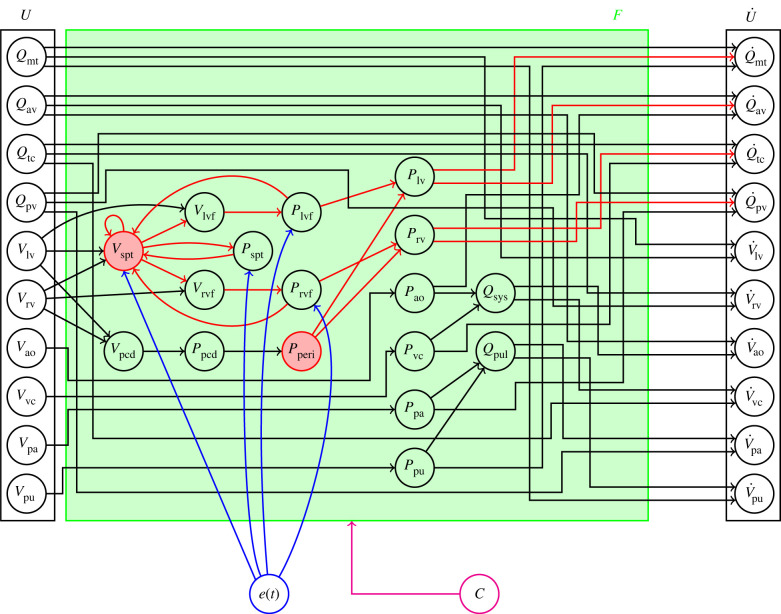
Graph of the lumped-parameter model of the cardiovascular system. The notation is consistent with equation (2.1). *U* is the set of the 10 state variables in the model, namely the six volumes of each of the elastic chambers (nodes) in figure 1 and the four flows where inertial effects are included. The relationship between *U* and its derivative ($\dot{U}$) is shown through the inter-dependencies among the intermediate variables in the model. The green region, which captures all of these inter-dependencies, represents the system equations (*F*) and consists of additional volumes, pressure and flows which are not state variables. The blue node and arrows represent the cardiac driver function (*e*(*t*)), and the magenta node and arrow represents the constant parameters of the model (*C*, defined in table 3). The red nodes and arrows within *F* represent the components of the model to be governed by a neural network, generating the structure of the hybrid neural ODE studied in this work (outlined in §2.2).

This model can be written as a system of ODEs, as follows:2.1
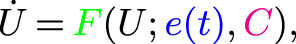
where *U* is the 10-dimensional vector of state variables, *F* is the set of system equations, *e*(*t*) is the cardiac driver function, *t* is time and *C* is the set of constant parameters used in the model. Figure 2 shows a graph of the model, where the dependencies between the state variables, the cardiac driver function and the other intermediate variables are shown. The colours of the letters in equation (2.1) match the corresponding colours in figure 2.

**Figure 3.**
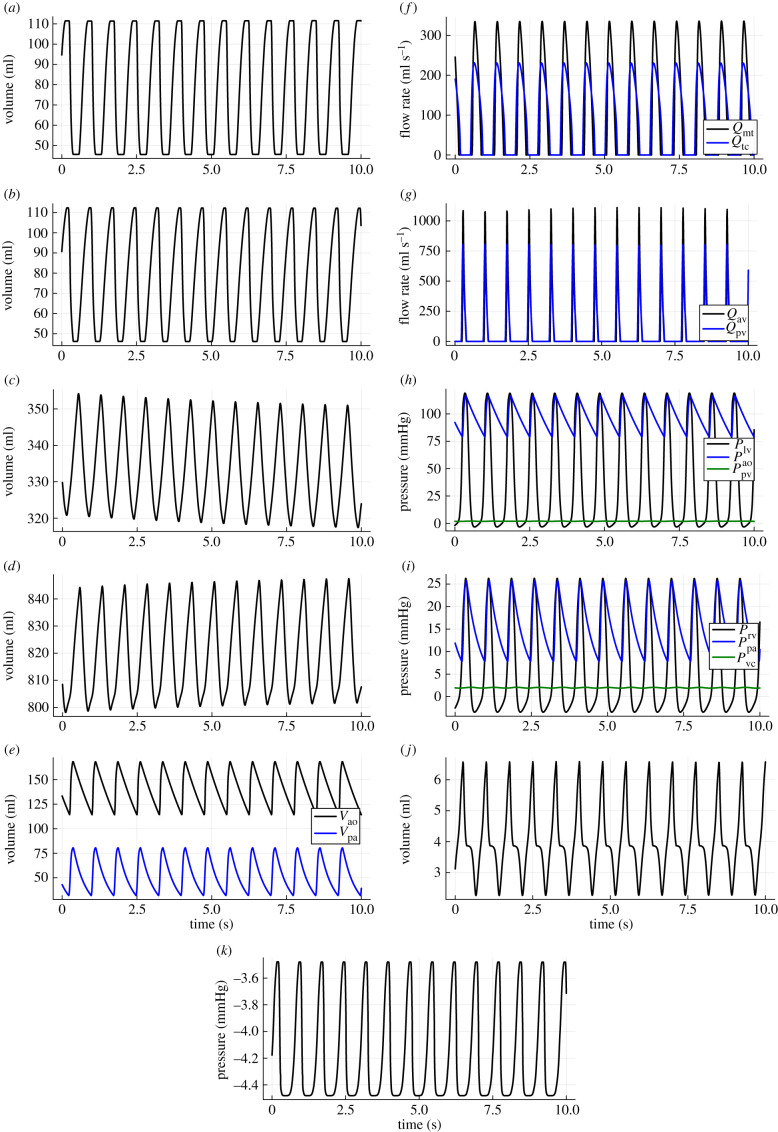
Synthetic time-series data generated from simulating the model outlined in figures 1 and 2. The model equations, the initial condition and the parameter values used in the simulation are given in appendix A. (*a*) Left ventricular volume (*V*_lv_), (*b*) right ventricular volume (*V*_rv_), (*c*) vena cava volume (*V*_rv_), (*d*) pulmonary vein volume (*V*_pu_), (*e*) aorta and pulmonary artery volumes (*V*_ao_, *V*_pa_), (*f*) mitral and tricuspid valve flow rates (*Q*_mt_, *Q*_tc_), (*g*) aortic and pulmonary valve flow rates (*Q*_av_, *Q*_pv_), (*h*) left ventricular, aortic and pulmonary vein pressures (*P*_lv_, *P*_ao_, *P*_pu_), (*i*) right ventricular, pulmonary artery and vena cava pressures (*P*_rv_, *P*_pa_, *P*_vc_), (*j*) septum free wall volume (*V*_spt_), (*k*) pericardium pressure (*P*_peri_).

This model (including *V*_spt_ as defined in algorithm 1) is simulated to generate the synthetic data that is used for training and validation. The full set of model equations, the initial condition and the set of constant parameters are given in appendix A. The simulation generates figure 3*a*–*k*, which show the temporal evolution of the 10 states, along with the septum-free wall volume and the pressure in the pericardium (the components which govern VI).

### 2.2. Hybrid neural ODE

**Figure 4.**
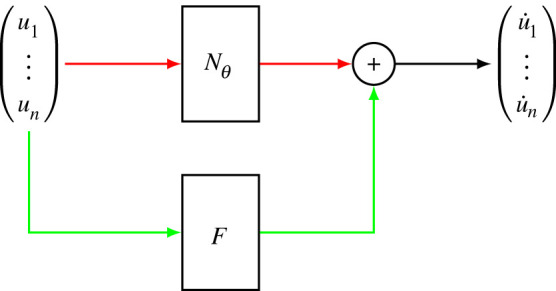
Hybrid neural ODE structure for a system with *n* dimensions. The state vector is passed to both a neural network $N_{\theta }$ (where *θ* is the set of weights and biases of the network) and a vector of mathematical operators *F*. The outputs are then combined to represent the derivative of the state vector. The green path represents a regular ODE, whereas the red path represents a neural ODE.

A hybrid neural ODE leverages partial mechanistic knowledge of the system to aid in the convergence of the neural network during the training process. As such, a hybrid neural ODE generally requires less training data than a neural ODE [30] (where the entire system of equations would be governed by a neural network). Figure 4 shows the structure of a hybrid neural ODE, highlighting how it is related to regular ODEs and neural ODEs.

As mentioned in §1, the volume of the septum free wall (a theoretical volume) is defined as the solution of a nonlinear equation with no analytic solution. The numerical solution of this equation is the main source of complexity in the model. The pressure in the septum is calculated as the difference between the ventricular pressures, and since the ventricles sit inside the pericardium, the pericardium pressure (*P*_peri_) also contributes to the VI. The original form of *P*_peri_ (as in [9]) is defined in equation (2.2)2.2
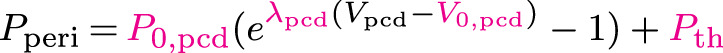


A hybrid neural ODE is constructed wherein *V*_spt_ and *P*_peri_ are replaced with a neural network. *P*_peri_ is selected to be approximated by a network, partly to examine the network’s ability to capture the dynamics of more than one component of the model, but also since it contributes to VI, and alternative models tend to leave out *V*_spt_ and *P*_peri_ together [13–16]. This architecture means that the nonlinear equation for *V*_spt_ need not be solved when simulating the model, while also allowing for a more parsimonious function with good predictive capabilities to be discovered via SR.

**Figure 5.**
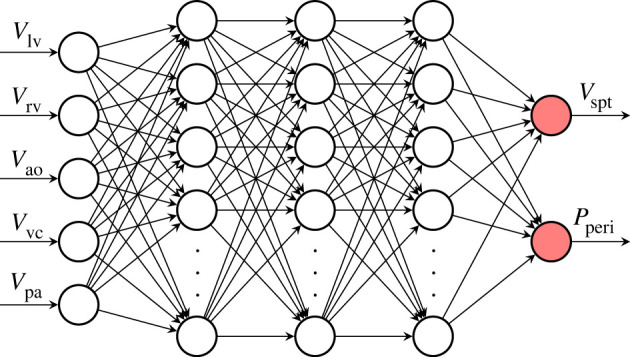
The neural network used in the hybrid neural ODE. The hidden layers consist of 10 neurons each, with ELU activation functions.


We use a fully connected neural network parametrized by a set of randomly initialized weights and biases. Although the model outlined in §2.1 has 10 state variables, the network takes in five volume states as inputs, namely [*V*_lv_, *V*_rv_, *V*_ao_, *V*_vc_, *V*_pa_]. This is because *V*_spt_ and *P*_peri_ depend on volumes rather than flows. Although they depend only on *V*_lv_ and *V*_rv_, using a network with just these two states as inputs was experimentally found to be less expressive. *V*_pu_ is omitted as an input to the network, as it was found that for the network architecture described, its inclusion hindered performance. There are three hidden layers of 10 neurons each, with exponential linear unit (ELU) activation functions. Finally, the network has two outputs, one for each of the components of the model (*V*_spt_ and *P*_peri_) it approximates. *V*_spt_ and *P*_peri_ are shown in red in figure 2 to highlight that they are governed by a neural network. Figure 5 shows the architecture of the network used in this work.

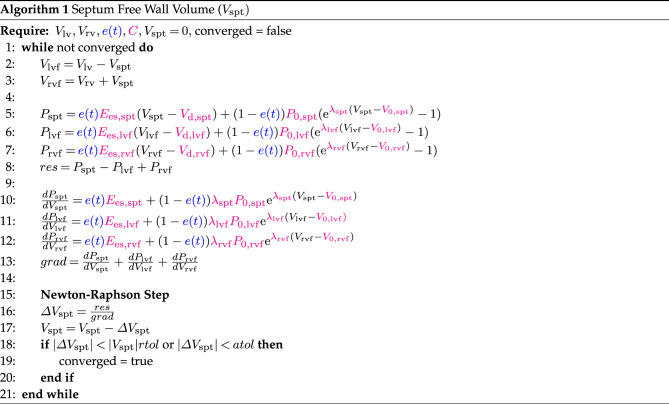


**Figure 6.**
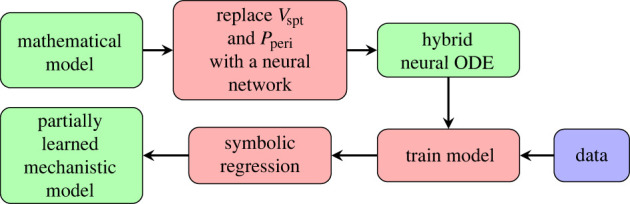
Flowchart of the method. The green cells represent the model at three different stages: the original mathematical model, the hybrid neural ODE and the partially learned model. The red cells represent the steps of the method: embedding a neural network within the model, training the hybrid model and implementing SR.

Figure 6 summarizes the method in a flowchart. The details of the training process and the implementation of SR are given in §§2.2.1 and 2.2.2, respectively.

#### 2.2.1. Training

Of the 10 s of synthetic data (hereafter referred to as ground truth) shown in figure 3, the first 0.3 s is selected for training. Since the dynamics are periodic, 0.3 s is selected to ensure the range of training data is less than one full period of the dynamics. This highlights an advantage of this type of hybrid modelling over black box models in that a short training range (and hence less data) is often sufficient to produce accurate generalizations, as the known components of the system are able to guide the model in its predictions. During each iteration of training, the hybrid neural ODE is simulated for 0.3 s to generate a prediction, which is then used in tandem with the ground truth to calculate a loss value. The loss function used in this work is mean squared error (m.s.e.) and is defined as

2.3 $$L=\frac{1}{N}\sum_{i=1}^{N}{\left(y_{i}-{\hat{y}}_{i}\right)}^{2},$$

where *L* is the loss value, *N* is the number of data points, *y* is the ground truth data and $\hat{y}$ is the prediction. In this work, a sampling rate of 0.01 is used (100 Hz), meaning a training range of 0.3 s corresponds to 30 data points per state variable, generating a training set of 300 data points. Hence, we have *N* = 300. The training then consists of minimizing this loss function by optimizing over the weights and biases of the network. The training is done using the Adam optimizer [31], which is a very common choice for optimization in machine learning because it is computationally efficient, easy to implement, and often works well on a wide range of tasks with little hyper-parameter tuning. A learning rate of 0.01 is used for 1000 iterations, followed by an additional 100 iterations with a learning rate of 0.0001.

The training process is carried out three times, at three different levels of noise. In particular, 0%, 2% and 5% of the standard deviation of the ground truth data is added to the ground truth in each case. In this work, an ensemble of 10 hybrid neural ODE models is used in the training process. This means that at each level of noise, the same hybrid neural ODE structure is trained 10 times. Due to the random initialization of the network parameters, each training process results in a different set of learned parameters and hence different predictions. The predictions of each of the 10 neural networks are then averaged. Averaging over the network outputs can increase predictive accuracy and allow for desired results to be achieved more consistently. There are many ways to average outputs, but in this work, a simple mean of the network predictions is taken. Furthermore, the random initialization of the network parameters results in a different rate of convergence for each training process. Therefore, for 0% noise, the selection criteria for the 10 models used in the ensemble was a maximum m.s.e. value of 1.0. For 2% and 5% noise, the maximum m.s.e. was selected to be 8.0 and 50.0, respectively.

#### 2.2.2. Symbolic regression

After training, the embedded neural network is regressed down from the high parameter space to mathematical expressions. This step results in a partially learned mechanistic model, which is more interpretable than the hybrid neural ODE and can often also help with achieving more accurate extrapolations. Here, an extrapolation refers to a simulation of the model beyond the time frame of the available data used to train the model (0.3 s). Although the extrapolations are expected to be superior to that of a black box model (due to the physical knowledge encoded in system equations), they can still lack some predictive accuracy, particularly if the training data only captures a very small portion of the dynamics. This motivates the use of a sparse regression technique in order to construct a partially learned, fully mechanistic model, with potentially improved extrapolation capabilities. Common choices for this regression step are SR and the sparse identification of nonlinear dynamical systems (SINDy) [32].

In this work, SR is used rather than SINDy due to the flexibility SR has in fitting nested expressions with real-valued arguments/exponents. SINDy, however, is an efficient alternative algorithm and a good choice when the user has some idea of the functional forms that may be required to explain the data. SR is a machine learning technique used to fit analytic expressions to data. It requires as input a set of unary operators (e.g. sin, cos, exp) and binary operators (e.g. addition, subtraction, multiplication, division). The function space defined by these operators is then searched in a ‘brute force’ manner via genetic programming. Processes such as mutations, crossovers and tournaments encourage a ‘survival of the fittest’ environment among different candidate expressions. For a more detailed description of SR, see [33,34].

Implementing SR involves setting the inputs to the network ([*V*_lv_, *V*_rv_, *V*_ao_, *V*_vc_, *V*_pa_]) as the inputs to SR, and setting the outputs of the network (the learned dynamics for *V*_spt_ and *P*_peri_) as the targets for SR. The Python package (with a Julia back-end) PySR [35,36] is used for the implementation of SR. We apply it to the averaged prediction of the networks in the ensemble. The details of the user-defined hyper-parameters for PySR used in this work are given in appendix B.

## 3. Results

**Figure 7.**
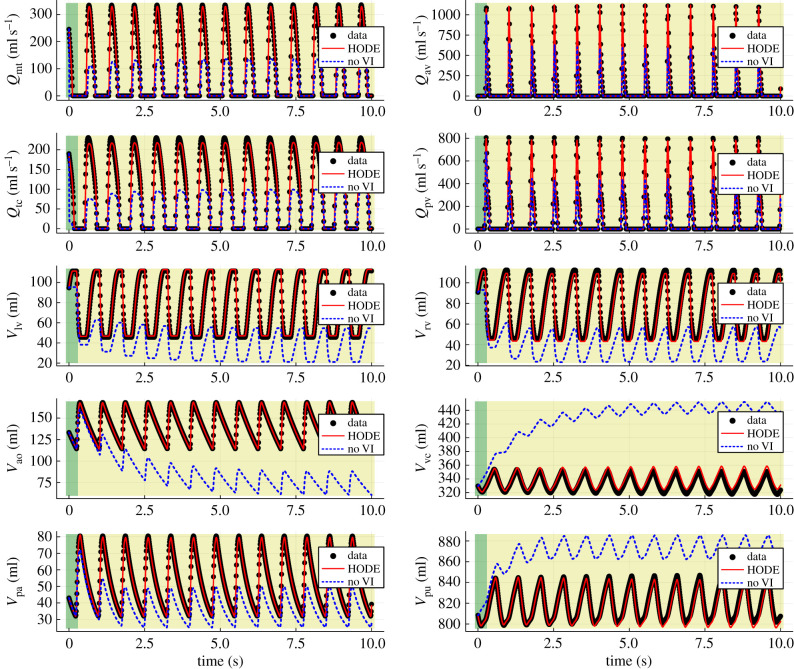
Extrapolation of the sixth hybrid neural ODE model (abbreviated to HODE in the figure labels) in the case of 5% noise. The black scatter points represent the ground truth data and the blue dashed curves represent the predictions of the model without VI. Ten plots are shown, one for each of the 10 state variables. The green region towards the left of each plot indicates the training range, while the yellow region is the extrapolation.

Upon training the 10 models in the ensemble, a single model is randomly selected and an extrapolation to 10 s is made to examine the model’s ability to generalize beyond the training data. In the case of 5% noise, model 6 was selected and the extrapolation is shown in figure 7. For comparison, the dynamics of the model without VI (by omitting algorithm 1 and equation (2.2) from the original model) are also shown.

Figure 7 shows that the hybrid neural ODE tracks the ground truth very well. The use of a small training range highlights the advantage of leveraging physical knowledge of the system over employing a black box approach, insofar as black box models typically struggle with extrapolations in this small data regime. However, the predictions of the hybrid neural ODE in figure 7 still deviate from the ground truth slightly in some cases, namely the *V*_vc_ and *V*_pu_ predictions.

**Figure 8.**
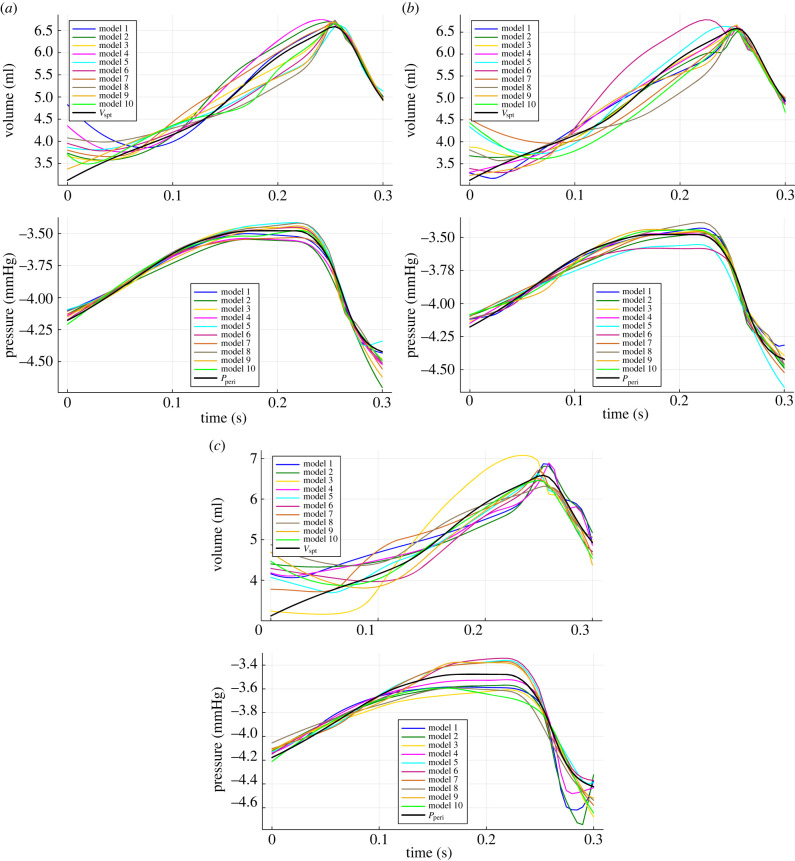
*V*_spt_ and *P*_peri_ predictions of the 10 neural networks in the ensemble, at each level of noise added to the ground truth data. (*a*) 0% noise, (*b*) 2% noise, (*c*) 5% noise.

To carry out SR, the hybrid neural ODE prediction is first generated in order to collect the temporal evolution of the five states [*V*_lv_, *V*_rv_, *V*_ao_, *V*_vc_, *V*_pa_], which the neural network takes as input. Next, the neural network is isolated from the hybrid neural ODE and the five states are given as inputs to the network. The two outputs of the network then correspond to the learned dynamics for *V*_spt_ and *P*_peri_. This is done at each level of noise. The learned dynamics of the 10 neural networks at each level of noise is shown in figure 8.

From figure 8, it can be seen that the neural networks generally approximate *P*_peri_ more accurately than *V*_spt_, although the overall climate of the dynamics is captured well in both cases. The curves generated from algorithm 1 and equation (2.2) are shown by the black curves. As mentioned in §2.2.2, the predictions of the 10 networks are averaged before applying SR. The input data for SR are the averaged inputs ([*V*_lv_, *V*_rv_, *V*_ao_, *V*_vc_, *V*_pa_]) of each of the 10 networks, while the target data for SR are the averaged outputs of the 10 networks. The functions learned via SR are given in table 1. The PySR algorithm assigns a score to a list of candidate functions, calculated based on a trade-off between accuracy and complexity [35]. In this work, the candidate function with the highest score was selected in each case.

**Table 1.** Learned functions via SR at each level of noise added to the ground truth data. The target data for SR was the averaged prediction (shown in red in figure 9).

term	noise	learned function
*P*_peri_	0%	(*V*_rv_(*V*_rv_ − 105.56)/3227.84) − 3.72
	2%	(1/*V*_pa_)(*V*_rv_ − 91.49) − 4.11
	5%	(1/*V*_pa_)(*V*_rv_ − 88.13) − 4.21
*V*_spt_	0%	(970.58/(*V*_ao_ − *V*_pa_)) + (12.83/(*V*_ao_ − *V*_rv_ + 2.54)) − 7.33
	2%	(1/(*V*_ao_ − *V*_pa_))(exp[(*V*_rv_/(*V*_pa_ − 10.05))] + 1080.36) − 8.63
	5%	exp[exp[(*V*_rv_/0.29(*V*_pa_)^2^)]]((*V*_vc_/(*V*_ao_ − *V*_pa_)) + 2.46)

**Figure 9.**
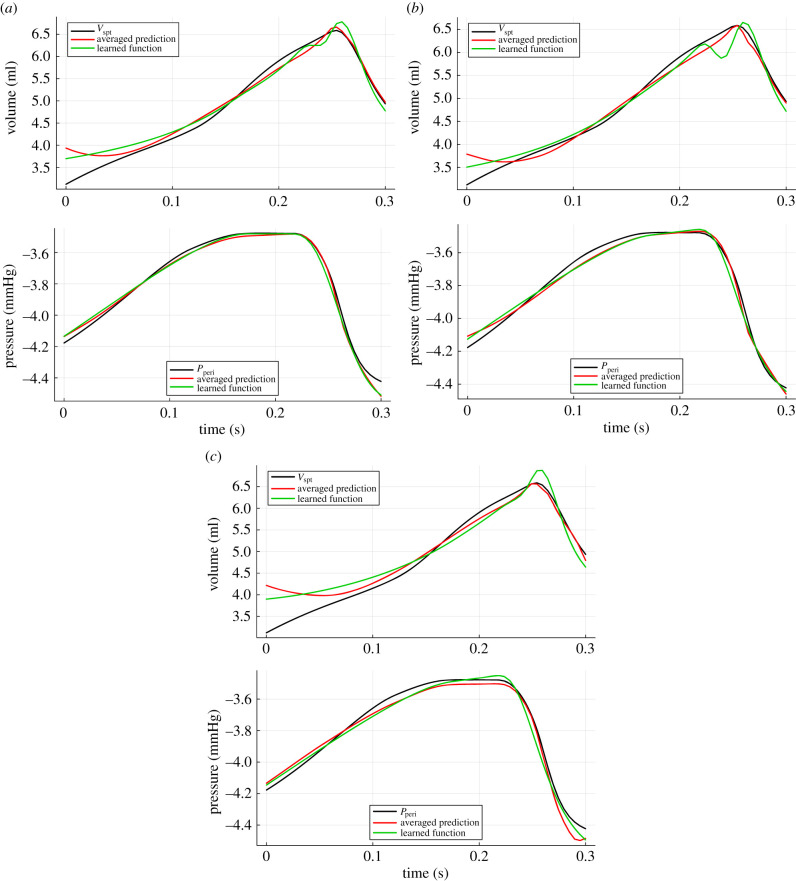
Averaged prediction of the 10 neural networks in the ensemble (red) and the dynamics of the corresponding learned functions via SR (green), at each level of noise added to the ground truth data. Table 1 shows the learned functions. (*a*) 0% noise, (*b*) 2% noise, (*c*) 5% noise.

Figure 9 shows the averaged outputs (red curves) of each of the networks in the ensemble, along with the corresponding learned function from table 1.

Equation (2.2) contains a term *P*_th_ which has a value of −4, as in table 3. Although the recovered functions for *P*_peri_ in table 1 are different at each noise level, they all contain a constant (−3.72, −4.11, −4.21) relatively close to –4. In figure 9, it is once again clear that *P*_peri_ is approximated more accurately than *V*_spt_. This is confirmed in table 2, where the m.s.e. between the averaged predictions of the networks (red curves in figure 9) and the original dynamics (black curves in figure 9), as well as the MSE between learned functions (green curves in figure 9) and the original dynamics is given. The values in table 2 under ‘learned MSE’ refer to the corresponding functions in table 1.

**Table 2.** The m.s.e. of averaged network outputs and learned functions, at each level of noise added to the ground truth data.

term	noise	averaged m.s.e.	learned m.s.e.
*P*_peri_	0%	0.000664	0.000988
	2%	0.000944	0.00124
	5%	0.00171	0.00158
*V*_spt_	0%	0.0467	0.0476
	2%	0.0313	0.0345
	5%	0.0912	0.102

It is worth highlighting that although the *P*_peri_ function from the original model (equation (2.2)) is within the function space defined by the unary and binary operators used in SR, table 1 shows that this function is not recovered. This is probably due to the short range of dynamics that is available and the little variation within this range (approx. between −4.4 and −3.5), since many functions can describe this curve. A further detail here is that as *V*_pcd_ is not one of the inputs to SR, the relationship *V*_pcd_ = *V*_lv_ + *V*_rv_ would also need to be recovered for equation (2.2). The true equation was only correctly recovered when applying SR directly to the ground truth (black curve in figures 8 and 9), using a training range of 0.5 s or longer.

**Figure 10.**
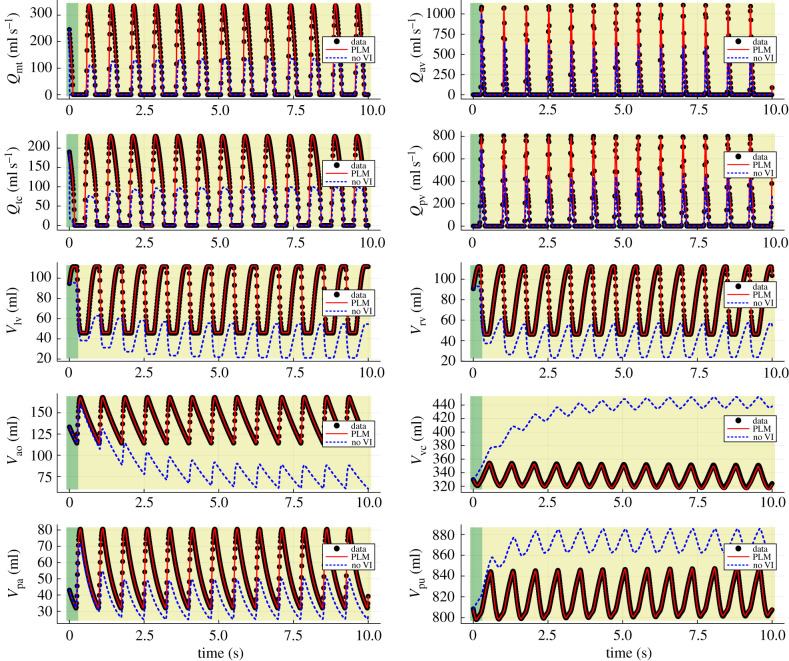
Extrapolation of the partially learned model (abbreviated to PLM in the figure labels) in the case of 5% noise (given in table 1). The black scatter points represent the ground truth data and the blue dashed curves represent the predictions of the model without VI. Ten plots are shown, one for each of the 10 state variables. The green region towards the left of each plot indicates the training range, while the yellow region is the extrapolation.

The embedded neural network in the hybrid neural ODE is then substituted with the recovered functions in the case of 5% noise, resulting in a partially learned mechanistic model. An extrapolation is subsequently made with this partially learned model, shown in figure 10. These predictions show a slight improvement from that of the trained hybrid neural ODE (figure 7). This improvement can also be quantified, as the root mean squared error (RMSE) between the hybrid neural ODE extrapolation in figure 7 and the ground truth is 6.393, while the RMSE between the extrapolation of the partially learned model and the ground truth is 3.680. As a result, we have a partially learned mechanistic model which captures VI in a more parsimonious fashion and has strong predictive capabilities.

## 4. Discussion

In this work, we show a novel means of modelling VI in the human CVS. We demonstrate the hybrid neural ODE’s ability (with robustness to some measurement noise) to discover alternative functions for *P*_peri_, and in the case of *V*_spt_, alternative and more parsimonious functions. The resulting partially learned mechanistic model of the CVS has good extrapolation capabilities. It is worth highlighting the primary reason for the different learned functions in table 1 is probably not the varying amounts of noise added to the ground truth data, but rather the randomness within the SR method. The randomly initialized populations of candidate functions often results in different learned functions upon completion.

A key rationale for the use of this methodology is to challenge modelling assumptions*.* This approach can either verify the structure of the model (by recovering the same equations as were originally proposed), or discover alternative functions to model the missing dynamics. This can be particularly useful within biomechanical modelling, as there is often uncertainty when modelling human physiology. The alternative approaches to modelling VI outlined in §1 motivate the use of this technique. We were able to recover functions for *V*_spt_ which are more parsimonious and interpretable than algorithm 1. The hybrid neural ODEs are more computationally expensive than the original model; however, partially learned models are more computationally efficient than the original model. These differences in simulation times only become significant when generating long ranges of synthetic data. For example, a 10 min simulation at a sampling rate of 0.01 takes 1.4569 s for the original model, 21.0804 s for a randomly selected hybrid neural ODE and 0.2960 s for the partially learned model.

An important clarification is that, while it is possible to forgo the use of a hybrid neural ODE and simply apply SR directly to the ground truth data, this is likely to be a much greater challenge. A short range of training data and a model with complex, nested ODEs (when expressed as functions of the states) greatly limits the possibility of SR to recover the governing equations. Alternative (more parsimonious) equations would be recovered, but this learned model would not provide any insight into VI, and would probably have problems when extrapolating.

Given the architecture of the hybrid neural ODE, it was expected that accurate extrapolations would be achieved when trained on only a small range of the dynamics, since the known equations provide information about the system. Given the periodic nature of the dynamics, the training range (0.3 s) was selected to be less than one period of the data. This is to highlight the advantage of the hybrid modelling approach, since extrapolations on periodic dynamics with a training range which captures more than one period can probably also be achieved with a black box model. This particular hybrid neural ODE was able to produce accurate extrapolations (similar to figure 7) with training ranges both significantly less than and greater than 0.3 s. These extremes, however, can be problematic for the SR step; a very short range of learned dynamics for *V*_spt_ and *P*_peri_ increases the challenge for SR to recover meaningful functions, while a long training range would require a significantly more expressive network in order to capture the *V*_spt_ and *P*_peri_ dynamics closely.

When employing an ensemble of networks, it is common to use varying architectures. The overarching idea is that with multiple architectures, the networks make errors in different regions of the parameter space, increasing the significance of the averaging step as the errors ‘cancel’ each other out. However, this work uses a single architecture for all 10 models in the ensemble, as outlined in §2.2. The reason for this is because of the proposed network’s superior performance over many alternative architectures.

A limitation of this approach is associated with the time required to complete the training process. During each iteration, the hybrid neural ODE must be simulated using an ODE solver. Therefore, if the model is computationally expensive (and/or a large range of the dynamics is simulated), training times can increase drastically. While this is not strictly a shortfall of the method itself, it has practical implications, especially if a more powerful network with more hidden layers is required. Another limitation is associated with the learned functions, in that the most accurate function recovered by SR does not guarantee the best performance of the corresponding partially learned model.

Potential avenues for future work include employing a Bayesian neural network structure. This way, training would be carried out once and generating multiple predictions of the hybrid neural ODE model would be done by sampling from the posterior distribution of the network parameters. Also, non-periodic dynamics can be generated if certain parameters in the model are allowed to vary with time (in a physiologically meaningful way), allowing for experiments to be carried out with varying lengths of the training range. We also plan to examine the performance of this method against real data, while simultaneously investigating whether the learned functions provide any physiological insight. This would involve gathering patient data on the 10 state variables, to be used as ground truth. The added challenge here is that alongside training the neural network, the parameters of the model would also need to be calibrated to fit the data, using a technique such as Bayesian history matching. Lastly, comparisons of the learned VI dynamics can be made with that of three-dimensional finite-element methods to further investigate the hybrid neural ODEs' predictive capabilities.

## Data Availability

The code developed for this work is available from the Zenodo digital repository: https://doi.org/10.5281/zenodo.10679484 [37].

## Appendix A. Cardiovascular model details

The system of 10 ODEs that define the original model as proposed in [9], including the intermediate variables that have not been defined in the main text, are given below.

$$\begin{array}{rl} & {\dot{Q}}_{\mathrm{mt}}=\{\begin{array}{cc} \frac{1}{L_{\mathrm{mt}}}[P_{\mathrm{pu}}-P_{\mathrm{lv}}-Q_{\mathrm{mt}}R_{\mathrm{mt}}],\; & \begin{array}{c} Q_{\mathrm{mt}}>0\;\mathrm{or}\;\\ P_{\mathrm{pu}}>P_{\mathrm{lv}}\; \end{array}\;\\ 0,\; & \;\mathrm{otherwise}\; \end{array}\\ & {\dot{Q}}_{\mathrm{av}}=\{\begin{array}{cc} \frac{1}{L_{\mathrm{av}}}[P_{\mathrm{lv}}-P_{\mathrm{ao}}-Q_{\mathrm{av}}R_{\mathrm{av}}],\; & \begin{array}{c} Q_{\mathrm{av}}>0\;\mathrm{or}\;\\ P_{\mathrm{lv}}>P_{\mathrm{ao}}\; \end{array}\;\\ 0,\; & \;\mathrm{otherwise}\; \end{array}\\ & {\dot{Q}}_{\mathrm{tc}}=\{\begin{array}{cc} \frac{1}{L_{\mathrm{tc}}}[P_{\mathrm{vc}}-P_{\mathrm{rv}}-Q_{\mathrm{tc}}R_{\mathrm{tc}}],\; & \begin{array}{c} Q_{\mathrm{tc}}>0\;\mathrm{or}\;\\ P_{\mathrm{vc}}>P_{\mathrm{rv}}\; \end{array}\;\\ 0,\; & \;\mathrm{otherwise}\; \end{array}\\ & {\dot{Q}}_{\mathrm{pv}}=\{\begin{array}{cc} \frac{1}{L_{\mathrm{pv}}}[P_{\mathrm{rv}}-P_{\mathrm{pa}}-Q_{\mathrm{pv}}R_{\mathrm{pv}}],\; & \begin{array}{c} Q_{\mathrm{pv}}>0\;\mathrm{or}\;\\ P_{\mathrm{rv}}>P_{\mathrm{pa}}\; \end{array}\;\\ 0,\; & \;\mathrm{otherwise}\; \end{array} \end{array}$$

$$\begin{array}{rl} & {\dot{V}}_{\mathrm{lv}}=\rho (Q_{\mathrm{mt}})-\rho (Q_{\mathrm{av}}),\\ & {\dot{V}}_{\mathrm{rv}}=\rho (Q_{\mathrm{tc}})-\rho (Q_{\mathrm{pv}}),\\ & {\dot{V}}_{\mathrm{ao}}=\rho (Q_{\mathrm{av}})-\rho (Q_{\mathrm{sys}}),\\ & {\dot{V}}_{\mathrm{vc}}=\rho (Q_{\mathrm{sys}})-\rho (Q_{\mathrm{tc}}),\\ & {\dot{V}}_{\mathrm{pa}}=\rho (Q_{\mathrm{pv}})-\rho (Q_{\mathrm{pul}}),\\ & {\dot{V}}_{\mathrm{pu}}=\rho (Q_{\mathrm{pul}})-\rho (Q_{\mathrm{mt}}), \end{array}$$

where

$$\begin{array}{rl} & Q_{\mathrm{sys}}=\frac{1}{R_{\mathrm{sys}}}(P_{\mathrm{ao}}-P_{\mathrm{vc}}),\\ & Q_{\mathrm{pul}}=\frac{1}{R_{\mathrm{pul}}}(P_{\mathrm{pa}}-P_{\mathrm{pu}}),\\ & P_{\mathrm{lv}}=P_{\mathrm{lvf}}+P_{\mathrm{peri}},\\ & P_{\mathrm{rv}}=P_{\mathrm{rvf}}+P_{\mathrm{peri}},\\ & P_{\mathrm{ao}}=E_{\mathrm{ao}}(V_{\mathrm{ao}}-V_{d,\mathrm{ao}}),\\ & P_{\mathrm{vc}}=E_{\mathrm{vc}}(V_{\mathrm{vc}}-V_{d,\mathrm{vc}}),\\ & P_{\mathrm{pa}}=E_{\mathrm{pa}}(V_{\mathrm{pa}}-V_{d,\mathrm{pa}})+P_{\mathrm{th}},\\ & P_{\mathrm{pu}}=E_{\mathrm{pu}}(V_{\mathrm{pu}}-V_{d,\mathrm{pu}})+P_{\mathrm{th}},\\ & V_{\mathrm{lvf}}=V_{\mathrm{lv}}-V_{\mathrm{spt}},\\ & V_{\mathrm{rvf}}=V_{\mathrm{rv}}+V_{\mathrm{spt}},\\ & V_{\mathrm{pcd}}=V_{\mathrm{lv}}+V_{\mathrm{rv}},\\ & e(t)=e^{-B_{1}{\left(\text{mod}\left(t,\frac{60}{\mathrm{HR}}\right)-C_{1}\right)}^{2}},\\ & \rho (x)=\left\{ \begin{array}{cc} x,\; & x\geq 0\;\\ 0,\; & \text{otherwise.}\; \end{array}\right. \end{array}$$

*V*_spt_, *P*_lvf_, *P*_rvf_ and *P*_spt_ are defined in algorithm 1, while *P*_peri_ is defined in equation (2.2). The values of the constant parameters (C) are given in table 3 and the initial condition of the system used in the simulation is given in table 4.

**Table 3.** Parameters for the closed-loop model of the cardiovascular system, as in [29]. These values were used for the simulation of the original cardiovascular model, the hybrid neural ODE and the partially learned model. EDPVR, end diastolic pressure volume relationship.

parameter	value
left ventricular end systolic elastance (*E*_es,lvf_)	2.8798 mmHg ml^−1^
left ventricular end systolic elastance (*E*_es,rvf_)	0.585 mmHg ml^−1^
end systolic septum elastance (*E*_es,spt_)	48.754 mmHg ml^−1^
aortic elastance (*E*_ao_)	0.6913 mmHg ml^−1^
vena cava elastance (*E*_vc_)	0.0059 mmHg ml^−1^
pulmonary artery elastance (*E*_pa_)	0.369 mmHg ml^−1^
pulmonary vein elastance (*E*_pu_)	0.0073 mmHg ml^−1^
mitral valve resistance (*R*_mt_)	0.0158 mmHg s ml^−1^
aortic valve resistance (*R*_av_)	0.018 mmHg s ml^−1^
tricuspid valve resistance (*R*_tc_)	0.0237 mmHg s ml^−1^
pulmonary valve resistance (*R*_pv_)	0.0055 mmHg s ml^−1^
pumonary circulation resistance (*R*_pul_)	0.1552 mmHg s ml^−1^
systemic circulation resistance (*R*_sys_)	1.0889 mmHg s ml^−1^
mitral valve inertia (*L*_mt_)	0.000077 mmHg s^2^ ml^−1^
aortic valve inertia (*L*_av_)	0.000121 mmHg s^2^ ml^−1^
tricuspid valve inertia (*L*_tc_)	0.000080 mmHg s^2^ ml^−1^
pulmonary valve inertia (*L*_pv_)	0.000149 mmHg s^2^ ml^−1^
unstressed left ventricular volume (*V*_d,lvf_)	0 ml
unstressed right ventricular volume (*V*_d,rvf_)	0 ml
unstressed septum volume (*V*_d,spt_)	2 ml
unstressed aortic volume (*V*_d,ao_)	0 ml
unstressed vena cava volume (*V*_d,vc_)	0 ml
unstressed pulmonary artery volume (*V*_d,pa_)	0 ml
unstressed pulmonary vein volume (*V*_d,pu_)	0 ml
zero-pressure left ventricular volume (*V*_0,lvf_)	0 ml
zero-pressure right ventricular volume (*V*_0,rvf_)	0 ml
zero-pressure septum volume (*V*_0,spt_)	2 ml
zero-pressure pericardium volume (*V*_0,pcd_)	200 ml
left ventricular EDPVR gradient (*P*_0,lvf_)	0.1203 mmHg
right ventricular EDPVR gradient (*P*_0,rvf_)	0.2517 mmHg
zero-volume septum pressure (*P*_0,spt_)	1.1101 mmHg
zero-volume pericardium pressure (*P*_0,pcd_)	0.5003 mmHg
left ventricular EDPVR curvature (*λ*_lvf_)	0.033 1/ml
right ventricular EDPVR curvature (*λ*_rvf_)	0.023 1/ml
septum EDPVR curvature (*λ*_spt_)	0.435 1/ml
pericardium EDPVR curvature (*λ*_spt_)	0.03 1/ml
heart rate (HR)	80 1/min
cardiac driver width (*B*_1_)	80
cardiac driver offset (*C*_1_)	30/80 s
thoracic cavity pressure (*P*_th_)	−4 mmHg

**Table 4.** Initial condition of simulation. These values were used for the simulation of the original cardiovascular model, the hybrid neural ODE and the partially learned model.

state	value
*Q*_mt_	245.581
*Q*_av_	0.0
*Q*_tc_	190.066
*Q*_pv_	0.0
*V*_lv_	94.681
*V*_rv_	90.730
*V*_ao_	133.338
*V*_vc_	329.780
*V*_pa_	43.012
*V*_pu_	808.458

## Appendix B. PySR details

Table 5 defines the hyper-parameters used in the implementation of PySR. The process begins with a given number of subpopulations (100), each containing a number (33) of randomly generated expressions from the function space defined by the binary and unary operators. Over a number of iterations (100), all the expressions are evolved and the ones which fit the target data best (m.s.e.) are more likely to survive. While we use 100 iterations as the stopping criteria for the process, alternative stopping criteria can be used, such as an amount of elapsed time or a desired fitness achieved.

**Table 5.** PySR hyper-parameters.

unary operators	{*e*}
binary operators	{+,−,÷,×}
functions per population	33
populations	1000
iterations	200
performance metric	m.s.e.
